# Supplementary material for: Spodoptera littoralis genome mining brings insights on the dynamic of expansion of gustatory receptors in polyphagous noctuidae
Source: G3 (Bethesda). 2022 Jun 2;12(8):jkac131. doi: 10.1093/g3journal/jkac131 (PMC9339325; doi:10.1093/g3journal/jkac131)
Supplement: jkac131_Supplementary_Data [file jkac131_supplementary_data.zip › Suppl/Data_S7_G3-2022-403383.docx]

BEB prob (ω>1) > 95%

BEB prob (ω>1) > 99%

CladeC

>SlituGR155

MFWHLTSTKKRPVKIYKHKILPYEEVLLNNVIEKDLQSILRPLNFMHHCFVCAKYTIRDNFITSNSLKYKLFGLICALFYRFFLLYDFGRNIYLYWNKFLTTLLIRLGYQNVTLAIGYFLIFFSNFLYCNDNVVLVVNIQNLIRTFKLERKHLNSFIIFNWFWVIFINFIFIFENYRNIITIDFRNFIFINVFSSIPSILYDINIVYAIFFVNLLKKTLKIMTKDLVRSSVRVSNSRSHWTEFYNSFANILETYNLFQKTFRLLIAFYSFFTISNSLLNVSIFITLGHDFNVIHITDILITFCSFVGRHILLLTLLCVQSEKLYAAFDESSYNSNLLCSLSESQRIVCRNIQRLYKASFKRFTVYGMFVIDVNFLFHLVAVISMYTIAQLQLILPPDE

>SlitGR217 (no sites)

CladeF

>SfruGR44

MDVILKDVLNYKSNVRKFINISISILRFAVGNYKKFNDSKYICFLAKLYCITVACCIIFRQIHVHGIFSLTKVIGRNPTRSRTRMKMLSNERYMERFSKGLSTCDAIMGFKDKSIMTEILFGISVSILIAKGLISLFWCYNISFELSTCVMMFSTEFNYLLHCVCQMTLYNRMGFIKKRLQSNLIHINIVGKDEIGRNVRVVRKCLGYYHNLLDNVQQLDTAMQVLVMALVISMMHVLAPSFITEMVNNNIDDIKSTLVTQMVRCSDKSLREELDTALQYIHRRPYKFVICGAVAVDGKLPFSIIGVCITYVILIKQFTHFVDVTS

>SfruGR49

MYHFKHFIQRHTPSNWFLKLLVILRLLLTNYTDISESRAKCYLLKFYCISIWFCFSLFYFYDVFARNAFGIYSLCVVEYTLCGIANFIGGDDFFFKFIRVIEINDRIIGFKKMSFFTKYLCVITVSNVTIRLFISITHAIVDPRPQYLFATVTFALLSTDINHIFNILIFSIIQNRMKQLQLFFKSIYIPVNISGRNEVEINIKSIRKGLLYYNNLLDNLRSISKSLQVLLFINWIIHCVKTLFLGFIIASLISRVHHKQLLGMMMEVMQSLIMISSPAIIASITANHVNDIKRMLSSKLLQTSDESLVFELETTLQYMTLRPFQXVVTLNIYLPFVVIGLCITYVVVGLQLSQIKI

>SlitGR44

MDGFLKNLLSYKSNVRKMYIITITVLRFVVGNYQKFNDSKRLCFFSKLYCITVACCLIYSHLNIHGLCFSPNLFAIEYSIYFVYYFITGQEYLMKFSKGLSTCDAIMNFKDMSILTEILFGISVSVLTAKYLTLYLYYNLSFDLSTCTVMFSTVFNYLLNYVLQMTLYNRMRIIKKCLQSNVIHINIVGKDQIGRNVRVVRKCLRYYHNLLDNIQELDITMQVLLSASLLCNIPQWIGGFSLSTDLWFNNVSGSVDLPMSMIHVMAPAIITELINNKIDEIKSTLVTQMIRCSDKSLREELETALQYIRRRPYKFVICGAVTVDGRLPISILSICITYVILTKQFIHFVDITI

>SlitGR49 (no sites)
