# Supplementary material for: Spodoptera littoralis genome mining brings insights on the dynamic of expansion of gustatory receptors in polyphagous noctuidae
Source: G3 (Bethesda). 2022 Jun 2;12(8):jkac131. doi: 10.1093/g3journal/jkac131 (PMC9339325; doi:10.1093/g3journal/jkac131)
Supplement: jkac131_Supplementary_Data [file jkac131_supplementary_data.zip › Suppl/Supplemental_Material_Legends_G3-2022-403383.docx]

**Supplementary figures**

**Figure S1** **Phylogeny of lepidopteran CSPs**

The dataset included amino acid sequences from *S. littoralis* (Noctuoidea, red), *S. litura* (Noctuoidea, green), *S. frugiperda* (Noctuoidea, orange), *B. mori* (Bombycoidea, blue) and *H. melpomene* (Papilionoidea, cyan). Sequences were aligned using MAFFT and the phylogenetic tree was reconstructed using PhyML. Midpoint rooting was used. Circles indicate nodes strongly supported by the likelihood-ratio test (aLRT>0.9). The scale bar represents 0.5 amino acid substitutions per site.

**Figure S2** **Phylogeny of lepidopteran OBPs**

The dataset included 53 amino acid sequences from *S. littoralis* (Noctuoidea, red), 53 sequences from *S. litura* (Noctuoidea, green), 53 sequences from *S. frugiperda* (Noctuoidea, orange), 44 sequences from *B. mori* (Bombycoidea, blue) and 43 sequences from *H. melpomene* (Papilionoidea, cyan). Sequences were aligned using MAFFT and the phylogenetic tree was reconstructed using PhyML. Subfamilies are indicated with different colors (yellow: Minus C subfamily, green: Plus-C subfamily, blue: PBP-GOBP subfamily). Midpoint rooting was used. Circles indicate nodes strongly supported by the likelihood-ratio test (aLRT>0.9). The red star indicates expansion in *Spodoptera*. The scale bar represents 0.5 amino acid substitutions per site.

**Figure S3 Genomic organization of the *Spodoptera* OBP genes.**

Scaffolds/chromosomes are represented in gray, with their numbers in italic. Gene names are indicated and their orientations are represented by the arrows.

**Figure S4 Phylogeny of lepidopteran IRs**

The dataset included amino acid sequences from *S. littoralis* (Noctuoidea, red), *S. litura* (Noctuoidea, green), *S. frugiperda* (Noctuoidea, orange), *B. mori* (Bombycoidea, blue) and *H. melpomene* (Papilionoidea, cyan). Sequences were aligned using MAFFT and the phylogenetic tree was reconstructed using PHYML. Colors indicate different categories of IRs (yellow: divergent IRs, grey: ionotropic glutamate receptors, orange: IR25a coreceptors. The tree was rooted using the iGluR clade. Circles indicate basal nodes strongly supported by the likelihood-ratio test (aLRT>0.9). The scale bar represents 0.5 amino acid substitutions per site.

**Figure S5** **Phylogeny of lepidopteran ORs**

The dataset included amino acid sequences from *S. littoralis* (Noctuoidea, red), *S. litura* (Noctuoidea, green), *S. frugiperda* (Noctuoidea, orange), *B. mori* (Bombycoidea, blue) and *H. melpomene* (Papilionoidea, cyan). Sequences were aligned using MAFFT and the phylogenetic tree was reconstructed using PHYML. The tree was rooted using the Orco clade (purple). Circles indicate basal nodes strongly supported by the likelihood-ratio test (aLRT>0.9). The scale bar represents 0.5 amino acid substitutions per site.

**Supplemental data**

**Data S1**

Genome sequencing data.

**Data S2**

Sequence logo of the GR conserved domain searched in S. littoralis, S. frugiperda and S. litura.

**Data S3**

CSP, OBP, IR and OR annotation in S. littoralis.

**Data S4**

GR annotation in S. littoralis, S. frugiperda and S. litura.

**Data S5**

Genome assembly metrics.

**Data S6**

TE-enrichment analysis around chemosensory genes.

**Data S7**

Positive selection analysis results.

**Data S8**

pLDDT scores for BmorGR9, SlitGR9, BmorGR66 and SlitGR15
