# Supplementary figures and images for: Spodoptera littoralis genome mining brings insights on the dynamic of expansion of gustatory receptors in polyphagous noctuidae
Source: G3 (Bethesda). 2022 Jun 2;12(8):jkac131. doi: 10.1093/g3journal/jkac131 (PMC9339325; doi:10.1093/g3journal/jkac131)

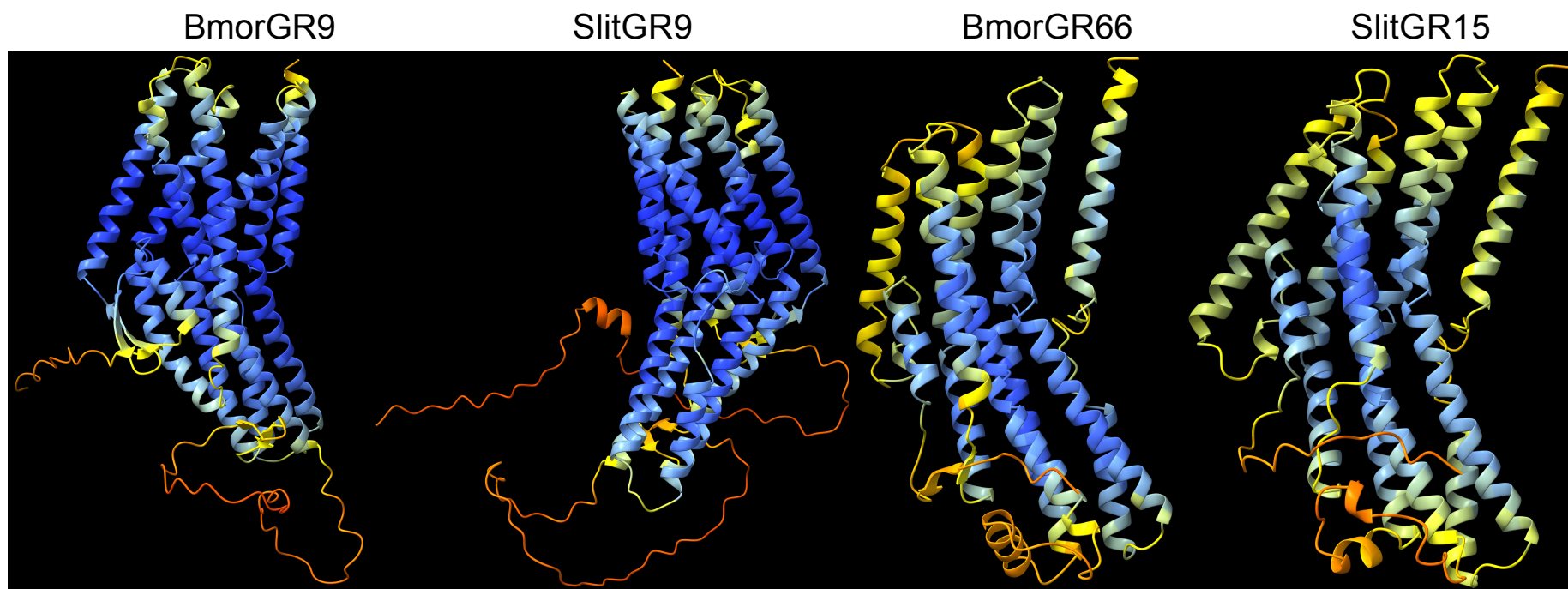

- Very high (pLDDT >90)
- Confident (90 >pLDDT >70)
- Low (70 >pLDDT >50)
- Very low (pLDDT <50)

Supplement: jkac131_Supplementary_Data [file jkac131_supplementary_data.zip › Suppl/Data_S8_G3-2022-403383.pdf]

Sequence logo analysis for all annotated GRs:

For SlitGRs:


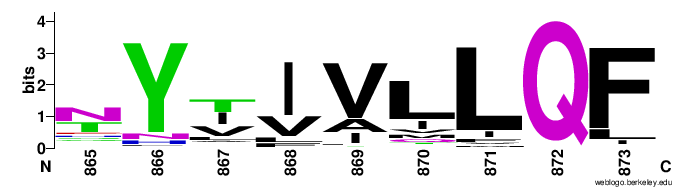


For SlituGRs:


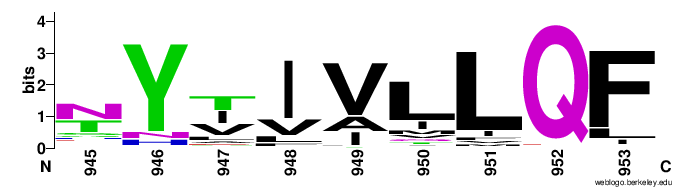


For SfruGRs:


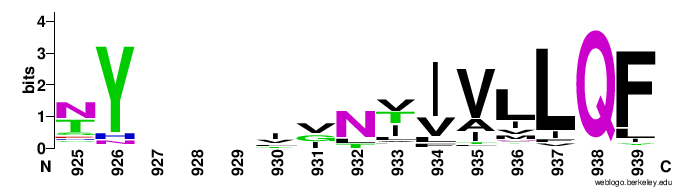

Supplement: jkac131_Supplementary_Data [file jkac131_supplementary_data.zip › Suppl/Data_S2_G3-2022-403383.docx]

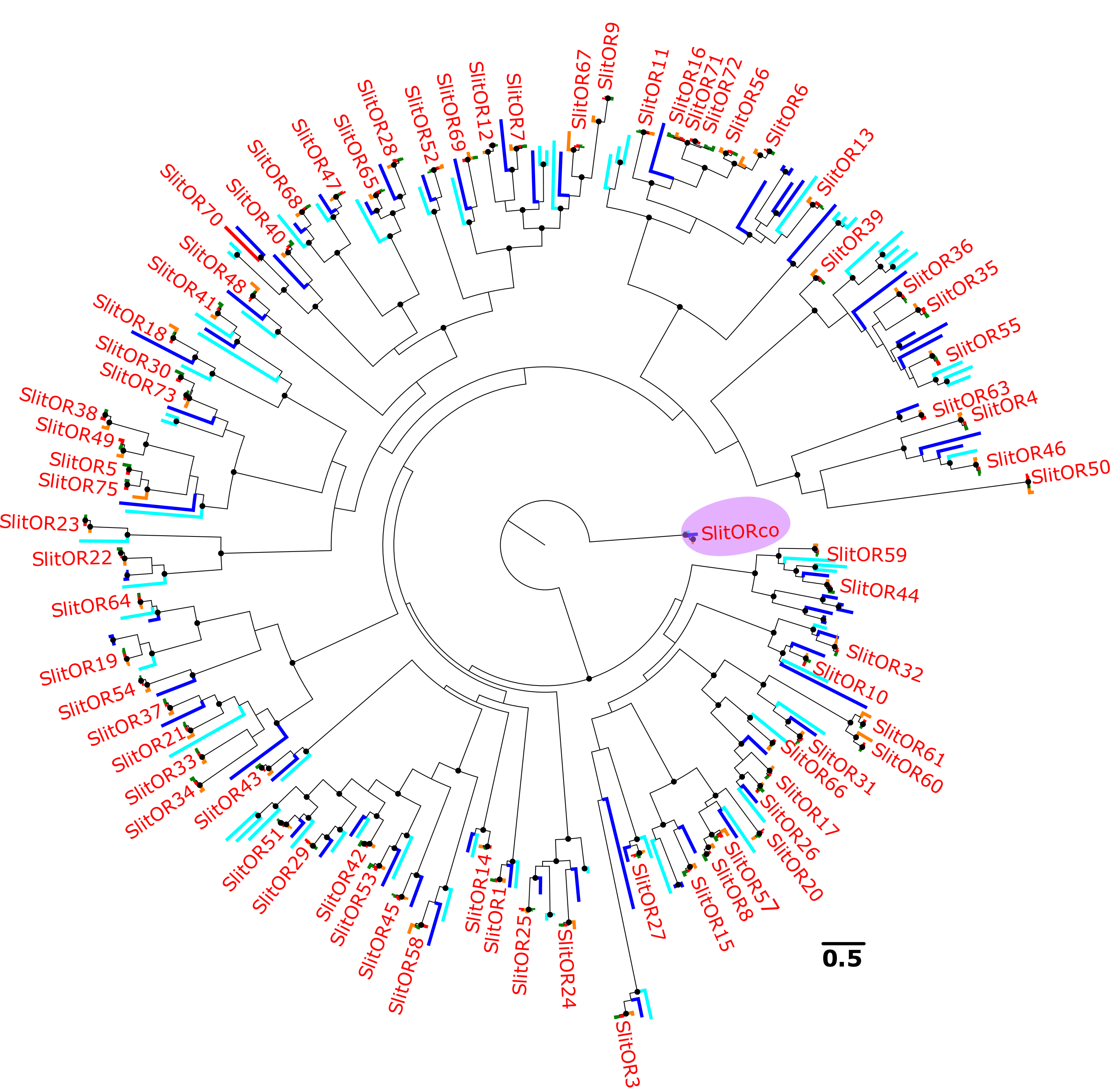

Supplement: jkac131_Supplementary_Data [file jkac131_supplementary_data.zip › Suppl/Figure_S5_G3-2022-403383.pdf]

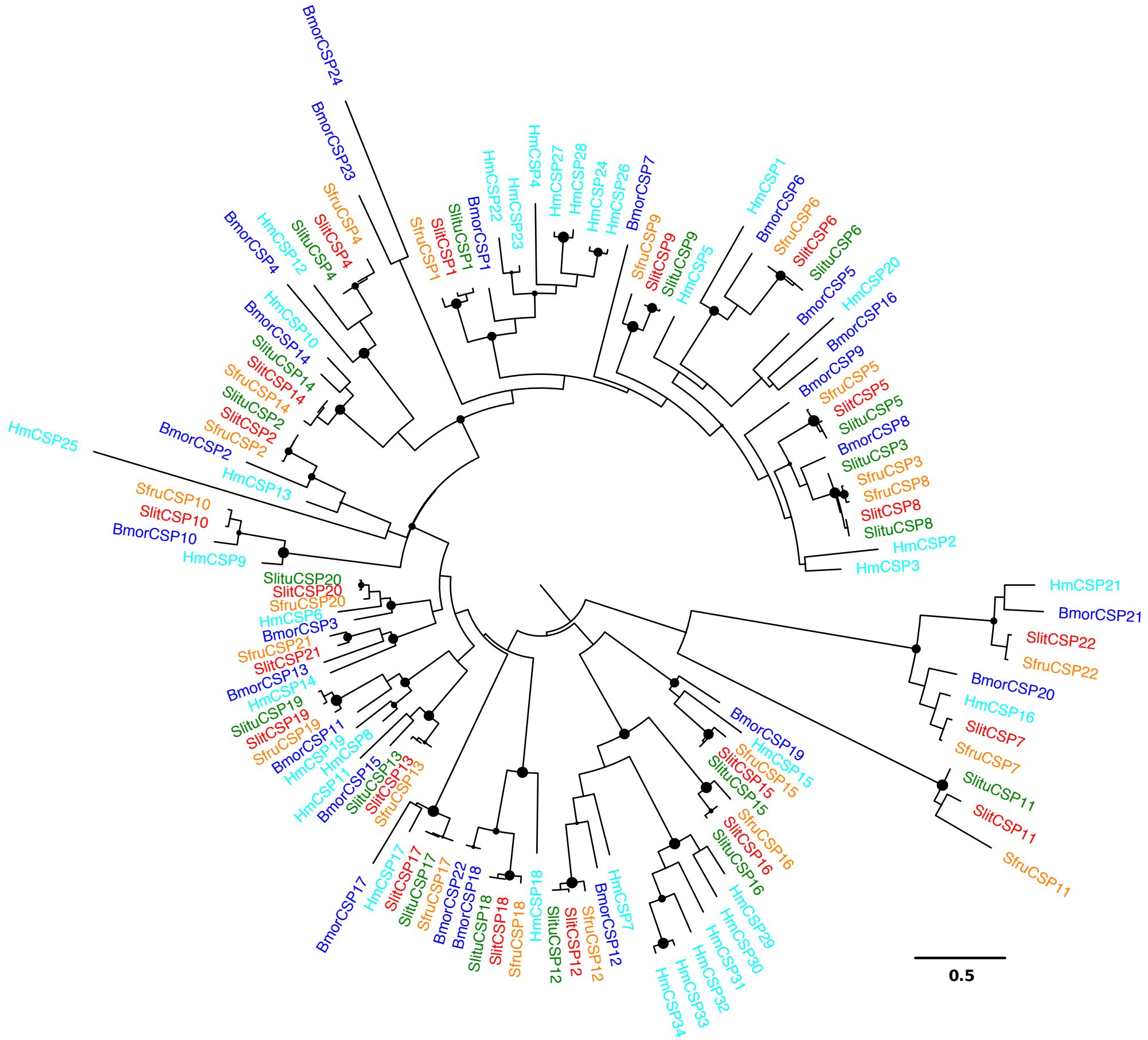

Supplement: jkac131_Supplementary_Data [file jkac131_supplementary_data.zip › Suppl/Figure_S1_G3-2022-403383.pdf]

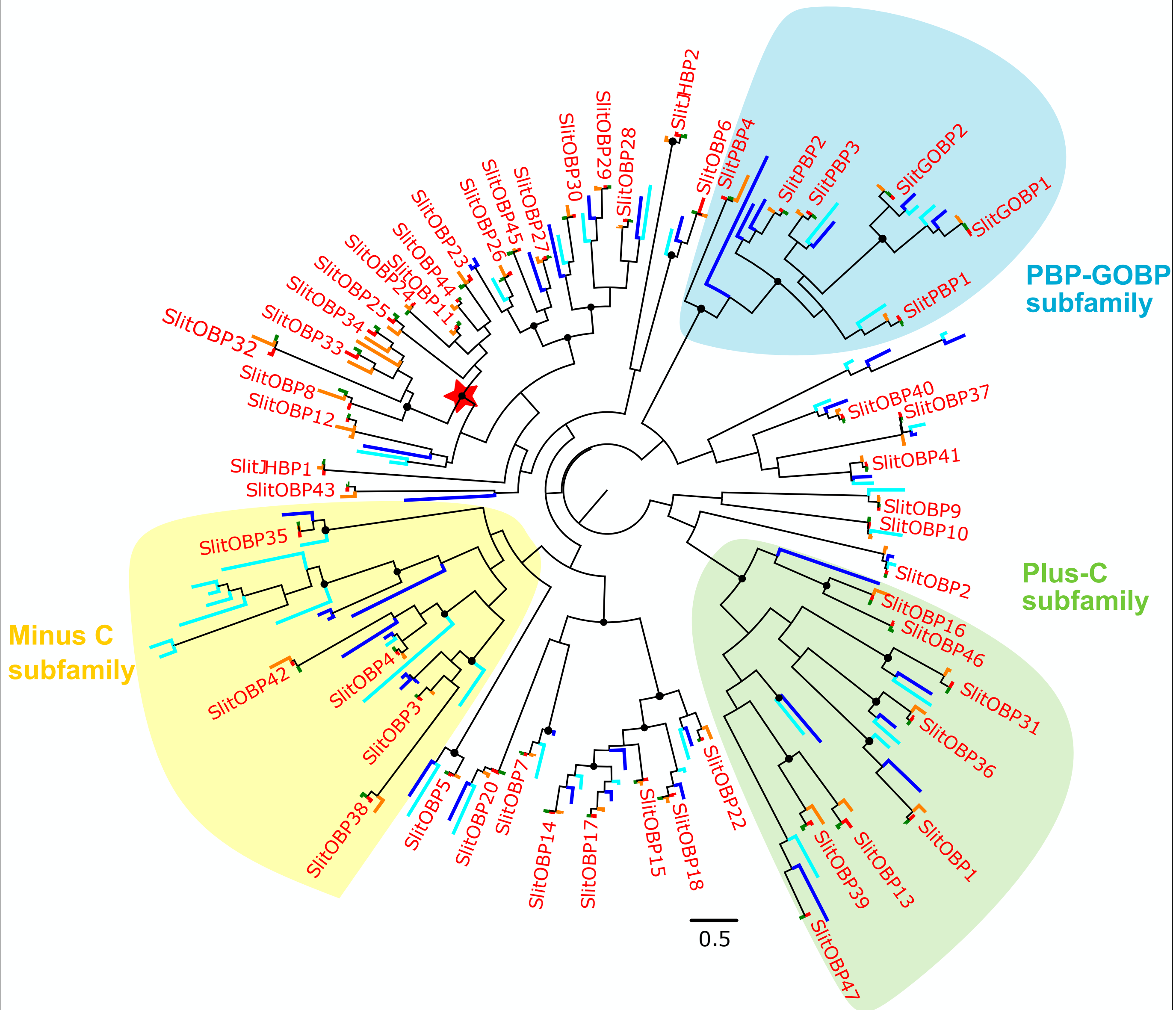

Supplement: jkac131_Supplementary_Data [file jkac131_supplementary_data.zip › Suppl/Figure_S2_G3-2022-403383.pdf]

*S. littoralis*

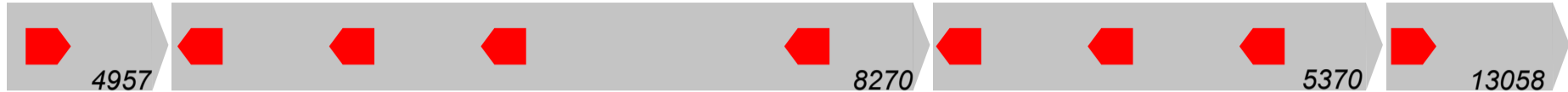

*S. litura*

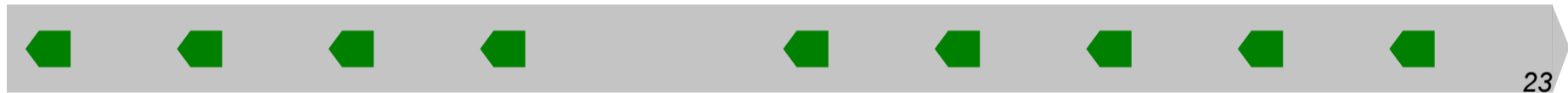

*S. frugiperda*

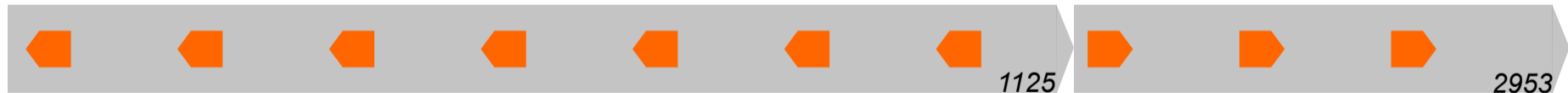

OBP44

OBP25

OBP32

OBP8

OBP21

OBP33

OBP34

OBP23

OBP11

OBP24

Supplement: jkac131_Supplementary_Data [file jkac131_supplementary_data.zip › Suppl/Figure_S3_G3-2022-403383.pdf]

## Divergent IRs

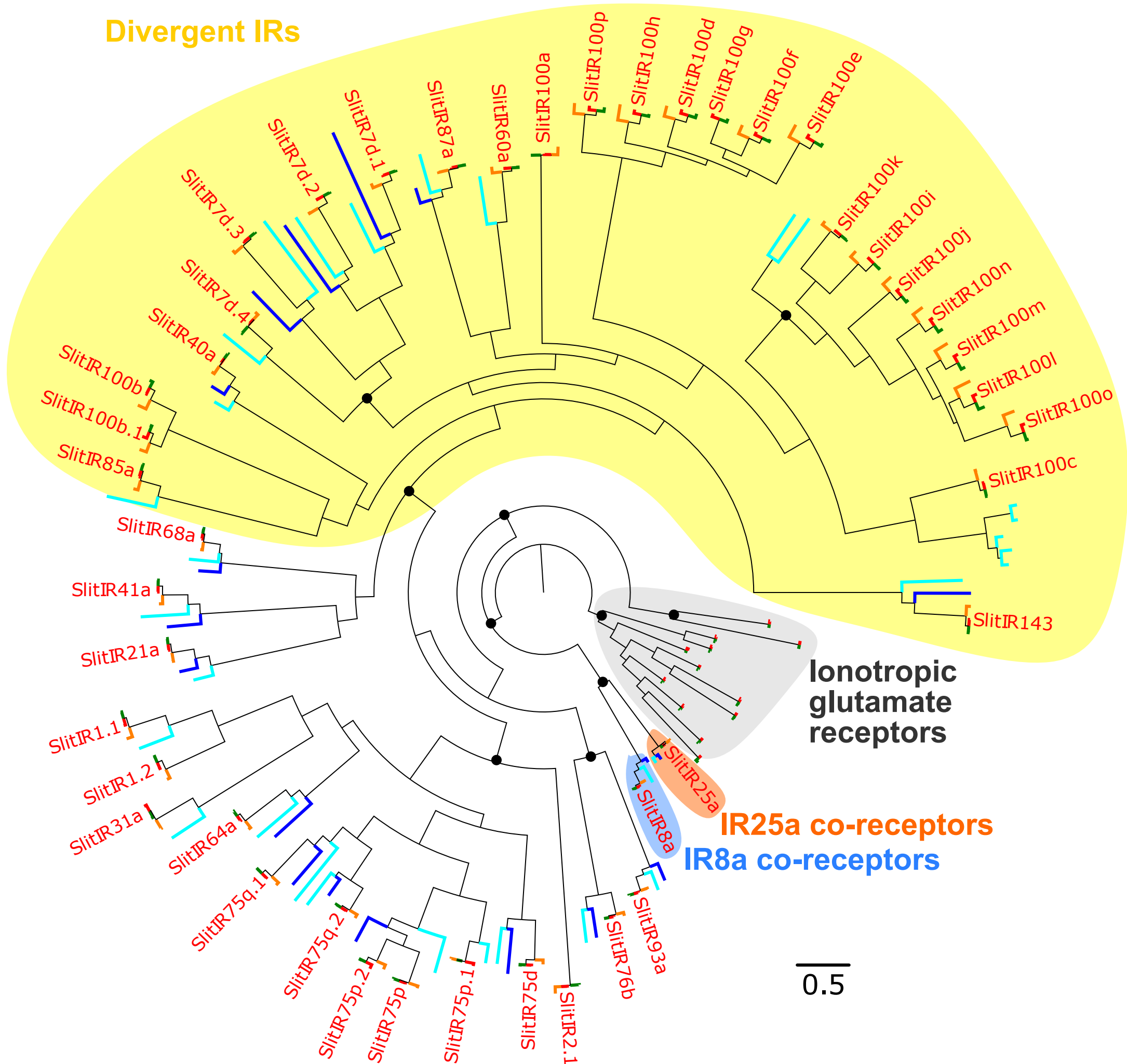

Supplement: jkac131_Supplementary_Data [file jkac131_supplementary_data.zip › Suppl/Figure_S4_G3-2022-403383.pdf]
